# Editorial: Fight against food waste: combating contamination and spoilage

**DOI:** 10.3389/fmicb.2023.1265477

**Published:** 2023-08-14

**Authors:** Laura Quintieri, Ok Kyung Koo, Oluwafemi James Caleb

**Affiliations:** ^1^Institute of Sciences of Food Production, National Research Council (CNR), Bari, Italy; ^2^Department of Food Science and Technology, Chungnam National University, Daejeon, Republic of Korea; ^3^Department of Food Science, Faculty of AgriSciences, Stellenbosch University, Stellenbosch, South Africa; ^4^African Institute for Postharvest Technology, Faculty of AgriSciences, Stellenbosch University, Stellenbosch, South Africa

**Keywords:** food safety, microbial contamination, biological control, fresh produce, *Listeria monocytogenes*

Over a third of all food produced (≈2.5 billion tons) is lost or wasted each year. Global food waste has an enormous environmental impact because it is a huge source of greenhouse gas emissions that is exacerbating the climate change crisis. Food wasted and/or lost becomes inaccessible to consumers, also contributing significantly to increased food insecurity in both developed and developing countries. In accordance with the United Nations Sustainable Development Goal of Agenda 2030, reducing food waste by improving food supply chain management represents one of the biggest challenges of this century (Ardra and Barua, 2022). Mitigation strategies such as recycling, alternative consumption models, and novel waste valorization technologies along with innovative solutions to reduce food spoilage are just a few examples of efforts by researchers and worldwide governments. Food spoilage can be caused by the growth of undesirable microorganisms which produce enzymes or metabolites with a biological function still unexplored in the food microbial communities but responsible for negative changes in the product (Quintieri et al., 2021).

This Research Topic on the “*Fight against food waste: combating contamination and spoilage*” aims to raise awareness on the issue of food waste and provide new research solutions to tackle global food loss and waste. We are pleased to note that our Research Topic has attracted contributions from many scientists and research teams across the globe with a total of 26 authors from around the world, including from Italy, the Republic of Korea, the Republic of South Africa, the UK, and USA. A total of nine submissions were expected, we received seven submissions, six of which were accepted (five original research articles and one review) for publication after rigorous peer review. The Research Topic covers the role of microorganisms in food spoilage, the risk posse by pathogenic species, and provides innovative methods and practices that can be employed to control microbial growth, extend shelf-life, and reduce losses along the food chain.

Several studies and reviews have postulated the most common causes of food waste, and the literature review by Karanth et al. provides an overview of the impact of microbial spoilage on food waste. The authors focused their review on microbial species involved in food deterioration and the route of microbial contamination across the food supply chain (pre-retail, retail, and consumer levels). Particular attention is given to the advancements of hurdle techniques and technologies [smart packaging, quantitative microbial spoilage risk assessments (QMSRAs), and edible anti-decay peels], as well as digital solutions (Internet of Things IoT) that could be crucial to the reduction food waste across the food value chain. The improvement of food product date labeling such as the use of “Best Before” and “Use by” via the adoption of smart labeling indicators of food freshness and safety such as the “After Opening labels” by Insignia Technologies and the RipeSense^®^ sensor label is also discussed.

Besides the use of smart food product labeling indicators to reduce waste along the value chains, the removal or control of food contaminates and spoilage microorganisms (pathogenic/non-pathogenic) by the use of antimicrobial agents is crucial. However, due to a major increase in the antimicrobial resistance of most pathogens to synthetic agents, there are growing concerns about environmental pollution with toxic chemical residues. The need for paradigm shifts from synthetic chemical compounds such as antimicrobial agents, the use of biological controls (e.g., bacteriophage GR1), natural plant extracts (e.g., essential oils, EOs), and chlorine-free treatments (ozone-assisted sanitizer) to manage food contaminants and pathogens were explored as current research trends. Kgang et al. report on the effects of lemon and lemongrass EOs against *Botrytis cinerea*, a necrotrophic fungal pathogen causing diseases in over 500 plants and a major cause of agricultural crop losses. Using enzymatic bioassays integrated with both gel-based and -free proteomics coupled with LC-MS/MS analysis. The authors established that the EOs treatments induced oxidative damage to the pathogen membrane lipids due to the accumulation of reactive oxygen species. Proteins linked to RNA helicase, transmembrane transporter, and antioxidant activities were above the set threshold in EO-treated *B. cinerea*. To develop an effective endolysin-based biocontrol agent, Choi and Kong isolated and characterized a novel *Geobacillus stearothermophilus*-infecting phage GR1 obtained from soil samples with a focus on its endolysin LysGR1 lytic activity. Choi and Kong demonstrated that *G. stearothermophilus* endolysin LysGR1 had a very broad bactericidal activity against tested strains of *G. stearothermophilus*, and foodborne pathogens including *Clostridium perfringens, Listeria monocytogenes*, and *Escherichia coli* O157:H7.

Another source of food contaminants and pathogens is in food processing and handling facilities on the surfaces of production equipment. Biofilm formation on the surface and within equipment increases the risk of cross-contamination with foodborne pathogens and exposure of food material to spoilage microorganisms. Cleaning-in-place (CIP) protocols are largely set up to minimize product cross-contamination and to ensure the safe removal of food material deposits. Sivri et al. investigate the formation of *Pseudomonas fluorescens* biofilm on pilot-scale simulating dairy products and assess the efficacy of ozone-based CIP to control the *P. fluorescens* biofilm. The authors demonstrate that ozone-based CIP eliminated *P. fluorescens* biofilm and reduced planktonic cells below the detection limit. To fully encourage the commercial adoption of natural plant extracts, bacteriophages, and alternatives to chlorinated CIP additional studies focused on safety issues along the food value chain are required.

*Listeria monocytogenes* has been an important foodborne pathogen due to its high mortality rate (20–30%), causing listeriosis in immunocompromised individuals. A shift in food implemented with listeriosis cases has been observed from milk and dairy products, deli meat, and other types of food, such as fruits (cantaloupe and stone fruits) and desserts (ice cream and profiteroles), indicating a broader range of potential sources (Desai et al., 2019). In this Research Topic, Moreira et al. evaluated the survival and growth of *L. monocytogenes* at either 4 or 13°C on fresh-cut produce and peeled rinds. The results showed that *L. monocytogenes* on fresh-cut pears increased slightly at 4°C, while a significant decrease was observed in kale, cauliflower, and broccoli. At 13°C, a significant growth on watermelons, cantaloupes, pears, papayas, and green bell peppers was observed. On the other hand, stable populations were observed on cantaloupe rinds at 24°C while the population on the outer surface of bell peppers decreased below detectable limits after 14 days at 4°C. These findings demonstrate that the survival behavior of *L. monocytogenes* on fresh-cut produce is influenced by the type of produce and the storage temperature.

The first outbreak case of *L. monocytogenes* was reported in 2018 in Korea. The outbreak strain was isolated from seasoned crab meat with bean sprouts, cold noodles, and salad. Lee et al. conducted a characterization of the *L. monocytogenes* strain from the patient through whole genome sequencing and compared it with the publicly available genomes of the same clonal complex. The strain belonged to ST224 and CC224 with specific genetic characteristics including antibiotic resistance genes, virulence genes, and a unique mutation in the LIPI-3. These findings are important for understanding the characteristics of the CC224 strains and their potential to cause listeriosis outbreaks. Both studies on *L. monocytogenes* in this Research Topic gave new insights into the emergence of *L. monocytogenes* contamination in food types and bacterial behavior during storage.

This Research Topic on the *Fight against food waste: combating contamination and spoilage* aims to raise awareness and provide research-based solutions to reduce global food loss and waste. We believe that by understanding the behavior and genetic characteristics of foodborne pathogens and spoilage microorganisms, we can make significant strides in achieving food security, safety, and sustainability. We hope that this Research Topic will encourage future research in the pursuit of the goal of zero hunger.

## Author contributions

LQ: Conceptualization, Validation, Visualization, Writing—original draft, Writing—review and editing. OK: Conceptualization, Validation, Visualization, Writing—original draft, Writing—review and editing. OC: Conceptualization, Validation, Visualization, Writing—original draft, Writing—review and editing, Supervision.
